# The impact of job fitness on depressive symptoms in Korean middle-aged and older population: a longitudinal study

**DOI:** 10.3389/fpubh.2024.1439058

**Published:** 2024-11-13

**Authors:** Il Yun, Seok-Hwan Jung, Doukyoung Chon, Jae-Hyun Kim, Jong Youn Moon

**Affiliations:** ^1^Department of Preventive Medicine, Gachon University College of Medicine, Incheon, Republic of Korea; ^2^Department of Health Administration, College of Health Science, Dankook University, Cheonan, Republic of Korea; ^3^Center of Public Health, Gil Medical Center, Gachon University of Medicine, Incheon, Republic of Korea; ^4^Artificial Intelligence and Big-Data Convergence Center, Gil Medical Center, Gachon University of Medicine, Incheon, Republic of Korea

**Keywords:** job fitness, job level, educational level, CES-D, depression, depressive symptoms

## Abstract

**Purpose:**

This study aimed to determine the impact of job fitness on depression and depressive symptoms in Korean middle-aged and older population.

**Methods:**

We collected data from the Korean Longitudinal Study of Aging (KLoSA), and performed a longitudinal analysis on 3,185 individuals with jobs at baseline. The dependent variable, depressive symptoms was measured by CES-D10 score, and the main variable of interest, job fitness was classified into nine groups according to job satisfaction and education level. To determine whether the estimate of depressive symptoms over the past week and depression changed over time, we applied the Generalized Estimating Equation (GEE) model.

**Results:**

In the fully adjusted model, the impact of job fitness on depression and depressive symptoms was statistically significant. Compared to those with a suitable job level and a medium educational level, those with a large gap between job and educational levels were more likely to develop depression and depressive symptoms. It was also found that satisfaction with job content was a crucial factor affecting depression in the middle-aged and older adults.

**Conclusion:**

Our findings demonstrated that job fitness significantly influenced depression, even after accounting for the effect of job satisfaction caused by a mismatch in job-education fitness. Since work demands and responsibility are difficult to quantify or qualify, job-education fitness could serve as a valuable tool to predict the extent of depression deterioration in individuals.

## Introduction

Depression or major depressive disorder (MDD), is a disease characterized by mood and mentality disorders associated with a negative impact on behavior, loss of interest, and emotional and physical problems which can lead to the degradation of an individual’s activity once enjoyed and ability to function at work and at home (1). Besides, depressive symptoms are positively correlated with the occurrence of suicide (2, 3) and chronic diseases (4, 5) and in terms of health scores, it is worse than in patients with chronic diseases (6).

The increase in depression occurrence is a global leading cause of disability and a great contributor to the global burden of disease (7). Many MDD occur after repetitive and persistent depressive symptoms, and indicators of the CESD scale include the experience of depressive symptoms (8). This, along with anxiety disorders, is an important catalyst for in not only depression but also schizophrenia, mental disorders, diabetes, and suicide (9). Depression is caused by biochemical, genetic, individual, and environmental factors as well as stress or changes in daily life and socioeconomic (SES) levels (10). Among various studies, it was found that low education levels, recent negative life events, and loneliness affected the overall life span and income; while, child abuse, and unhealthy aspects including pain, BMI and number of chronic diseases seemed more strongly associated with depression and depressive syndrome in older ages (11, 12). It was also known that physical activity and exercise volume have a significant impact on depressive symptoms and mental health (13, 14). In addition, financial dependence and work environment were the most important factors involved in the onset of depressive syndrome (12).

Based on several studies on depression as above, there is a lot of various view on depression. Many studies also focused on the impact of relationship between occupation and mental health such as job stress (15), depression and depressive symptoms in specific occupations (16–18), and working conditions on mood disorder (19). There were also studies focused on the importance of educational level found that the area, the learning content and focus, afterschool activities were all factors affecting not only the individuals’ knowledge but also their personality and mental health (20–22). Freeman et al. (23) also used combined scores of socioeconomic variables to measure their effect on the onset of depression.

Based on the knowledge available regarding job and education as single factors, we decided to analyze the effect of job education fitness on major depressive disorder. Job fitness refers to the degree of equilibrium between job and worker’s characteristics such as personality, knowledge, skills, and job performance (24). Currently, the health care industry focuses more on the job, work environment’ stress and specific health risk than job fitness. However, in the economic sector, the mismatch in job fitness is identified as a potential threat to the fourth industrial revolution and as a factor affecting workers’ wages, performance, productivity, and job satisfaction (25, 26). Job fitness should also be considered important in the health and medical fields because factors involved in the onset of depression, such as job satisfaction (27), wages (28), and job stress (15) can occur individually or together when work demands and personal attributes, such as education level, are not aligned. There are various forms of job fitness such as a genuine and apparent mismatch (29), real and formal mismatch (30), educational and skill mismatch (31).

In this study, we decided to study job-education mismatches because of two reasons. First, individuals tend to have better (or worse) jobs depending on their educational level (32), which can be measured through subjective responses comparing job level to educational level. Furthermore, depending on the position, workplace, and personal characteristics of the CEO, the job requirements can vary. Because job requirements cannot be quantified or qualified, disequilibrium between these factors has a negative impact on stress, satisfaction and so on. Second, generally, the standard of education depends on each country and is defined by a graduation system. For this reason, we think many countries can apply this mismatch of job-education by replacing the variables according to the country’s education system. In this study, the job satisfaction, which is correlated with fitness, is included as covariates in the analysis in order to identify the pure effect of job-education fitness on depression. In addition, we attempted to control as much as possible potential confounding factors that could affect depressive symptoms. Through these considerations, comparison between the job and the actual education level are utilized to present academic grounds on what job fitness means for depression and depressive symptoms by utilizing a large panel data.

## Materials and methods

### Data source

We used the Korean Longitudinal Study of Aging (KLoSA) data to identify and analyze the effect of job fitness in relation to educational level on depression. The first wave of KLoSA occurred in 2006 and contained participants’ data for community-dwelling Koreans who were 45 years old and older. Since the first wave of KLoSA in 2006, follow up interviews were held every 2 years until 2018. Participants were selected randomly using a multistage, stratified probability sampling design based on the sampling frame. A sampling frame was set up by geographical areas and housing types across Korea to create a nationally representative sample. The data are composed of eight categories: population, family, health status, employment, income, assets, subjective expectations, and quality of life. This original KLoSA study population included South Korean adults 45 years or older who lived in 15 large administrative areas. In the first baseline survey, 10,254 individuals dispatched in 6,171 households (1.7 individuals per household) were surveyed using the computer-assisted personal interviewing method. In 2008, the second survey followed up with 8,688 subjects, representing 86.6% of the original panel. In 2010, the third survey followed up with 7,920 subjects, representing 81.7% of the original panel. In 2012, the fourth survey followed up with 7,486 subjects, representing 80.1% of the original panel. In 2014, the fifth survey followed up with 7,029 subjects, representing 79.2% of the original panel and 920 new subjects included as a new panel. The size of the total sample was 7,949 subjects and represented 80.4% of the sample maintenance rate. In 2016, the sixth survey followed up with 7,490 subjects (including 872 new participants of the fifth), representing 79.6% of the sample maintenance rate. Finally, the seventh survey followed up with 6,940 subjects (including 804 new participants of the fifth), representing 78.8% of the sample maintenance rate. Due to data limitation, we excluded the first baseline survey which lacks the CES-D10 score variable.

Therefore, our study used data from six surveys of KLoSA (2nd to 7th). To identify and analyze the impact of job fitness and educational level on depression, unemployed and economically inactive research participants and unpaid family volunteers were excluded from the study. Participants who had no information about variables or non-response or rejection of responses were also excluded from the study. The general characteristics of the subjects were analyzed using the second survey data from the KLoSA and resulted in the inclusion of a total of 3,185 participants, 2,138 male and 1,047 female workers.

### Independent variables

#### Actual educational level

The actual educational level of the subjects was categorized into three groups depending on their graduation level: “Low” for middle school or lower, “Medium” for high school, and “High” for college or higher.

#### Subjective conception of current job levels

In order to identify the subjective conception of current job levels according to the level of education, five-step measures were used according to the question, “How do you think the level of your occupation is compared to your level of education?”: (1) Very low; (2) Low; (3) Suitable; (4) High; (5) Very high. In this study, we classified responses into three categories: low (very low and low), Suitable, and high (high and very high).

#### Job fitness

To determine the job fitness, we intended to evaluate the difference between the current job level (relative to educational level) and the actual educational level. The difference between the two variables was classified as follows: Current Job level (compared to Educational level) – Actual Educational Level: Low-Low (≤middle school), Low-medium (high school), Low-High (≥college), Suitable-Low (≤middle school), Suitable-Medium (high school), Suitable-High (≥college), High-Low (≤middle school), High-Medium (high school), and High-High (≥college).

### Dependent variables

#### The depressive symptoms over the past week

The occurrence of depressive symptoms was assessed for 7 days before the interview. Responder were asked about their level of depressive symptoms over the past week. This was classified as follows: (1) Less than a day; (2) 1–2 days; (3) 3–4 days; (4) 5–7 days. In this study, we classified responses into two categories: No (less than a day), Yes (1–7 days).

#### Modified CES-D10 score and depression

In this study, we applied the Modified Center for Epidemiologic Studies Depression Scale (CES-D) to measure depressive symptomatology (8). The CES-D scale remains one of the most widely used tools in the field of psychiatric epidemiology (33, 34). According to KLoSA’s data, 10 questions out of 20 in CES-D were selected for subjects of the 2nd to 4th wave according to the Andresen form (35). After the 5th wave, data were obtained according to the Boston form (36). The CES-D 10 scores were summed up according to the Korean version of CES-D. According to KLoSA, and the preceding study, the depression symptoms were measured with the total score of CES-D10 (37–39). Modified CES-D scale is total 10 points and we set 4points to the cutoff value of depression.

### Control variable

The covariates of this study are listed based on previous literature as follows (40–42): satisfaction with the job content, gender, age, marital status, self-rated health, number of chronic diseases, smoking status, drinking status, regular physical activity, and the modified CES-D10 form.: “satisfaction with the job content” was measured by a question that “I am satisfied with my job” and it was classified as follows: very satisfied, satisfied, not satisfied and not satisfied at all. “Gender” was categorized into two groups: male, female. “Age” was categorized into four groups: 45–54, 55–64, 65–74, and ≥ 75 (75 or older). “Marital status” categorized participants as married, separated, divorced, and ‘single’ (never married). For “Self-rated health,” participants were asked to rate their health status on a five point Likert scale (1 corresponding to very good and 5 to very bad). In this study, we categorized “Self-rated health” into three groups: good (very good & good), moderate (normal), bad (bad & very bad). The “Number of chronic diseases” is a covariate that refers to the number of chronic illnesses in participants such as high blood pressure, diabetes, cancer, chronic pulmonary disease, liver disease, cardiovascular disease, cerebrovascular disease, mental illnesses, and arthritis. Subjects were categorized into three groups: 0, 1–2, ≥3 (three or more). “Smoking status” referred to nonsmoker who never smoked, former smoker, or smoker and “Drinking status” to drinker, former drinker, and nondrinker. “Regular physical activity” was defined by regular exercise (more than once a week) and participants were categorized as yes or no.

### Statistical analysis

In this study, we performed the chi-square test, the Generalized Estimating Equation (GEE) model, and a clustered model. For the GEE model, we used the *proc genmod* with link: *log and logit, distribution: normal and binary* to determine whether the estimate of depressive symptoms over the past week and depression changed over time. The regression coefficient was used to estimate both the change in depression symptoms and independent variables. Depression and the depressive symptoms over the past week where the outcomes in all GEE models. Statistical analysis was performed on a total of five periods each considered as a separate situation regardless of the participants’ number in the study itself. Covariates of interest from all subjects were added to the model to determine their effects on reporting depression. Satisfaction with one’s job is an important covariate, and the pattern of the association between current job suitability and depressive symptoms may vary accordingly, so a subgroup analysis by job satisfaction was performed to confirm this. All statistical analyses were performed using the SAS statistical software package, version 9.4 (SAS Institute, Inc., Cary, NC, United States). All statistical tests were two-tailed with a significance of *p* < 0.05.

## Results

### Baseline general characteristics of participants

The baseline general characteristics of participants are shown in Table 1. Of the total 3,185 subjects, 1,121 people had depression. The group with the most depression, was found for the group with a high degree of job level and a low degree of educational level (≤middle school). The baseline prevalence of depressive symptoms over the past week was 26.9% (857 participants) among all participants. We also observed a pattern with the job content satisfaction, the lower the satisfaction, the higher depression rate and the percentage of subjects with depressive symptoms in the past week. The depression rate was 14.8% for the most satisfied group and 51.7% for the most dissatisfied group. The proportion of participants with depressive symptoms in the past week was 9.1% (eight participants) in the most satisfied group.

**Table 1:** General characteristics of subjects included for analysis.

	Total		Depression			Depressive symptom over the past week		
	*N*	%	Yes	%	*p* value	Yes	%	*p* value
Current job suitability (Educational level) − Educational level					<0.0001			<0.0001
Low- ≤ middle school	234	7.4	102	43.6		78	33.3	
Low-high school	290	9.1	94	32.4		69	23.8	
Low- ≥ college	111	3.5	32	28.8		18	16.2	
Suitable- ≤ middle school	1,284	40.3	547	42.6		442	34.4	
Suitable-high school	892	28.0	255	28.6		182	20.4	
Suitable- ≥ college	341	10.7	74	21.7		56	16.4	
High- ≤ middle school	13	0.4	8	61.5		8	61.5	
High-high school	10	0.3	3	30.0		0	0.0	
High- ≥ college	10	0.3	6	60.0		4	40.0	
Satisfaction with current work contents					<0.0001			<0.0001
Very Agree	88	2.8	13	14.8		8	9.1	
Agree	1,941	60.9	589	30.4		441	22.7	
Disagree	1,009	31.7	443	43.9		350	34.7	
Not at all	147	4.6	76	51.7		58	39.5	
Gender					<0.0001			<0.0001
Male	2,138	67.1	682	31.9		516	24.1	
Female	1,047	32.9	439	41.9		341	32.6	
Age					<0.0001			<0.0001
45–54	1,403	44.1	417	29.7		321	22.9	
55–64	1,080	33.9	365	33.8		268	24.8	
65–74	589	18.5	265	45.0		208	35.3	
≥ 75	113	3.6	74	65.5		60	53.1	
Self-rated health					<0.0001			<0.0001
Good	1,682	52.8	404	24.0		278	16.5	
Moderate	1,099	34.5	458	41.7		360	32.8	
Bad	404	12.7	259	64.1		219	54.2	
Marital status					<0.0001			<0.0001
Married	2,784	87.4	902	32.4		676	24.3	
Separated/divorced	374	11.7	209	55.9		172	46.0	
Single	27	0.9	10	37.0		9	33.3	
Income					<0.0001			0.000
Yes	1,561	49.0	493	31.6		1,187	76.0	
No	1,624	51.0	628	38.7		1,141	70.3	
Smoking status					0.253			0.225
Never smoker	1,724	54.1	629	36.5		481	27.9	
Former smoker	499	15.7	167	33.5		120	24.1	
Current smoker	962	30.2	325	33.8		256	26.6	
Drinking status					0.001			0.022
Drinker	1,802	56.6	586	32.5		453	25.1	
Former drinker	262	8.2	104	39.7		83	31.7	
Never	1,121	35.2	431	38.5		321	28.6	
Number of chronic disease					<0.0001			<0.0001
0	1,943	61.0	569	29.3		418	21.5	
1–2	1,147	36.0	493	43.0		385	33.6	
≥ 3	95	3.0	59	62.1		54	56.8	
Regular physical activity(≥ 1 a week)					<0.0001			<0.0001
Yes	1,053	33.1	284	27.0		214	20.3	
No	2,132	66.9	837	39.3		643	30.2	
Total	3,185	100.0	1,121	35.2		857	26.9	

*Hypertension, diabetes, cancer, chronic obstructive pulmonary disease, liver disease, cardiovascular disease, cerebrovascular disease, mental illness, and arthritis.

### Impact of job fitness on depression

In the fully adjusted model (Table 2), the association between current job fitness and depression was statistically significant, with the following odds ratio (OR) predicting increased depression: OR = 3.421 (*p* < 0.0001) for individuals with a high job level and a high educational level, OR = 2.194 (*p* = 0.008) for individuals with a high job level and a low educational level, and OR = 1.696 (*p* < 0.0001) for individuals with a low job level and a low educational level as compared to the group with a suitable job level and a medium educational level. We also found that the decrease in the degree of satisfaction was associated with an increase the OR of depression. Participants classified as “not satisfied at all” in job content obtained the OR of 3.029 (*p* < 0.0001) compared to individuals with very satisfying job content (Table 2).

**Table 2:** The adjusted impact of job suitability on depression.

	Depression			
	OR	95%CI		*p* value
Current job suitability (Educational level) − Educational level				
Low- ≤ middle school	1.696	1.451	1.983	<0.0001
Low-high school	1.316	1.137	1.523	0.000
Low- ≥ college	1.238	1.009	1.519	0.041
Suitable- ≤ middle school	0.998	0.904	1.102	0.974
Suitable-high school	1.000			
Suitable- ≥ college	1.026	0.898	1.174	0.703
High- ≤ middle school	2.194	1.226	3.923	0.008
High-high school	2.059	1.147	3.697	0.016
High- ≥ college	3.421	1.845	6.341	<0.0001
Satisfaction with current work contents				
Very Agree	1.000			
Agree	1.403	1.062	1.853	0.017
Disagree	2.596	1.953	3.450	<0.0001
Not at all	3.029	2.159	4.250	<0.0001
Gender				
Male	1.000			
Female	1.080	0.969	1.205	0.164
Age				
45–54	1.000			
55–64	1.058	0.962	1.163	0.247
65–74	1.084	0.960	1.223	0.194
≥ 75	1.296	1.088	1.544	0.004
Self-rated health				
Good	1.000			
Moderate	1.510	1.389	1.642	<0.0001
Bad	3.225	2.841	3.661	<0.0001
Marital status				
Married	1.000			
separated/divorced	1.860	1.663	2.080	<0.0001
Single	1.645	1.146	2.363	0.007
Income				
Yes	1.000			
No	1.146	1.062	1.237	0.000
Smoking status				
Never smoker	1.135	1.009	1.276	0.035
Former smoker	1.000			
Current smoker	1.192	1.062	1.339	0.003
Drinking status				
Drinker	1.000			
Former drinker	1.146	1.014	1.296	0.029
Never	1.004	0.918	1.099	0.924
Number of chronic disease				
0	1.000			
1–2	1.041	0.959	1.130	0.333
≥ 3	1.321	1.107	1.576	0.002
Regular physical activity (≥ 1 a week)				
Yes	1.000			
No	1.100	1.011	1.197	0.026
Year				
2008	1.390	1.230	1.570	<0.0001
2010	1.379	1.221	1.556	<0.0001
2012	1.221	1.078	1.383	0.002
2014	1.117	0.990	1.260	0.073
2016	1.000			

### Impact of job fitness on depressive symptoms

In the fully adjusted model (Table 3), the association between current job fitness and depressive symptoms measured for a week was statistically significant. The following odds ratio (OR) predicted increased depressive symptoms: OR = 3.493 (*p* < 0.0001) for individuals with a high job level and a low educational level, OR = 2.261 for individuals with a high job level and a high educational level and OR = 1.515 (*p* < 0.0001) for individuals with a low job level and a low educational level as compared to the group of individuals with a suitable job level and a medium educational level. We also found that the decrease in the degree of satisfaction was associated with an increase in the odds ratio of depressive symptoms. Participants in the group classified as “not satisfied at all” in job content obtained an OR value of 2.537 (*p* < 0.0001) compared to the group with very satisfying job content (Table 3).

**Table 3:** The adjusted impact of job suitability on depressive symptom in the past week.

	Depressive symptom in the past week			
	OR	95%CI		*p* value
Current job suitability (Educational level) − Educational level				
Low- ≤ middle school	1.512	1.285	1.779	<0.0001
Low-high school	1.316	1.127	1.536	0.001
Low- ≥ college	1.179	0.946	1.469	0.142
Suitable- ≤ middle school	1.059	0.953	1.176	0.286
Suitable-high school	1.000			
Suitable- ≥ college	0.991	0.858	1.146	0.906
High- ≤ middle school	3.484	1.947	6.234	<0.0001
High-high school	1.500	0.790	2.848	0.215
High- ≥ college	2.193	1.124	4.279	0.021
Satisfaction with current work contents				
Very Agree	1.000			
Agree	1.392	1.027	1.888	0.033
Disagree	2.391	1.754	3.261	<0.0001
Not at all	2.529	1.762	3.630	<0.0001
Gender				
Male	1.000			
Female	1.094	0.976	1.227	0.122
Age				
45–54	1.000			
55–64	0.996	0.901	1.103	0.945
65–74	0.982	0.865	1.116	0.786
≥ 75	1.146	0.958	1.371	0.137
Self-rated health				
Good	1.000			
Moderate	1.587	1.451	1.736	<0.0001
Bad	3.592	3.156	4.089	<0.0001
Marital status				
Married	1.000			
separated/divorced	1.735	1.547	1.946	<0.0001
Single	2.128	1.483	3.055	<0.0001
Smoking status				
Never smoker	1.161	1.025	1.316	0.019
Former smoker	1.000			
Current smoker	1.158	1.023	1.311	0.021
Drinking status				
Drinker	1.000			
Former drinker	1.224	1.077	1.390	0.002
Never	1.046	0.952	1.151	0.349
Number of chronic disease				
0	1.000			
1–2	1.059	0.971	1.155	0.197
≥ 3	1.368	1.143	1.637	0.001
Regular physical activity (≥ 1 a week)				
Yes	1.000			
No	1.133	1.036	1.240	0.006
Year				
2008	0.984	0.866	1.117	0.799
2010	0.999	0.881	1.134	0.992
2012	0.875	0.768	0.997	0.044
2014	0.999	0.883	1.131	0.992
2016	1.000			

### Subgroup analysis by job satisfaction

As shown in Table 4, in subgroup satisfied with their job, the association between job fitness and depression and depressive symptoms over past week was not statistically significant for the individuals with a high job level and high education level (OR = 1.933, *p* = 0.478, OR = 3.438, *p* = 0.186) and for the individuals with a low job level and a high educational level (OR = 0.744, *p* = 0.088, OR = 0.901, *p* = 0.566).

**Table 4:** The adjusted impact of job suitability on depression/depressive symptom in the past week by satisfaction with current work contents.

		Depression				Depressive symptom in the past week			
		OR	95%CI		*p* value	OR	95%CI		*p* value
	Current job suitability (Educational level) − Educational level								
Groups dissatisfied with their current work contents	Low- ≤ middle school	2.659	2.170	3.258	<0.0001	2.221	1.796	2.748	<0.0001
	Low-high school	1.903	1.577	2.297	<0.0001	1.833	1.502	2.237	<0.0001
	Low- ≥ college	1.571	1.218	2.026	0.001	1.322	0.998	1.753	0.052
	Suitable- ≤ middle school	1.110	0.985	1.252	0.088	1.117	0.982	1.270	0.092
	Suitable-high school	1.000	1.000	1.000		1.000	1.000	1.000	
	Suitable- ≥ college	1.083	0.931	1.258	0.301	1.018	0.865	1.198	0.833
	High- ≤ middle school	3.113	1.311	7.391	0.010	4.261	1.802	10.073	0.001
	High-high school	1.916	0.897	4.093	0.093	1.525	0.650	3.577	0.332
	High- ≥ college	3.836	1.994	7.377	<0.0001	2.075	0.993	4.339	0.052
Groups satisfied with their current work contents	Low- ≤ middle school	0.830	0.648	1.062	0.138	0.872	0.676	1.125	0.293
	Low-high school	0.697	0.552	0.882	0.003	0.782	0.611	1.001	0.051
	Low- ≥ college	0.744	0.530	1.045	0.088	0.901	0.633	1.284	0.566
	Suitable- ≤ middle school	0.733	0.612	0.878	0.001	0.891	0.739	1.075	0.229
	Suitable-high school	1.000	1.000	1.000		1.000	1.000	1.000	
	Suitable- ≥ college	1.045	0.754	1.448	0.792	1.068	0.759	1.504	0.706
	High- ≤ middle school	1.362	0.626	2.964	0.436	2.513	1.146	5.509	0.021
	High-high school	1.855	0.687	5.010	0.223	1.245	0.460	3.368	0.666
	High- ≥ college	1.933	0.313	11.935	0.478	3.438	0.551	21.432	0.186

## Discussion

In this study, we identified the relevance between job fitness and depression by including the job level and the actual level of education of participants over six periods of KLoSA’s panel data (survey 2 in 2008 to 7 in 2018). According to our study, analyses of CES-D10 scores and depressive symptoms showed that low, high job levels, compared to a suitable job level, showed a negative impact on two indicators of depression. And the both showed a higher degree of depression at a High job level. We found that the probability of experiencing depressive symptoms increased as the job content became less satisfactory. Many similar study (26, 43, 44) suggest that job or education has a meaningful effect on depression from a socioeconomic perspective. In this study, we analyzed the association between job fitness (compared to educational level) and depression. In contrast to prior studies, we suggested that job-education fitness was one of the factors of depression. And our preliminary data showed that job-education fitness affect depression and that series of depression problems can occur when individuals think their job level is inappropriate with their level of education. This study supports previous research with additional evidence for the impact of job and education level on depression. This means that research with additional evidence for the impact of job and educational level on depression. This means that research analyzing depression must be considered through multi-dimensioned variables. Based on the results of Nordin et al. (45), an income penalty may result from a mismatch between the level of job and education. Additionally, Lee et al. (46) stated that income and depression were highly related. They showed that individuals with low income regardless of sex were more likely to develop Major Depressive Disorders (MDD) and that low income can serve as a significant risk factor of MDD. This is supported by our data showing how the gap between job and education levels affected depression in mismatched groups. According to these studies, job and educational levels may directly or indirectly affect depression because of income penalty, low income and so on. The design of the study did not allow us to find any difference between age-specified groups. Participants included in the surveys were all middle-aged or older individuals still engaged in economic activities. Our data were consistent with other study (11) showing that older people are more likely affected by socioeconomic factors including job-fitness.

Many studies investigating the effect of job stress on depression have demonstrated its adverse effect on mental health (15) in all areas regardless of certain ages, professions, nationalities, etc. (47–49). The Center for Disease Control and Prevention in the United States defined job stress as a physical and emotional adverse reaction that occurs when the requirements of work do not match capabilities, resources or requirements of the worker, and emphasized the working conditions and the personal characteristics of the worker. In this study, the results that can able to influence depression based on differences in the educational level of individuals and the job level required if individuals at work, we provided data to support our hypothesis regarding the impact of existing “Job Stress,” individual’s ability and characteristics on the occurrence of depression.

Similarly to the study of Lee et al. (50), the CES-D score, depression level, and proportion of depressive symptoms were consistent with the high values obtained for the group of subjects with a high job level. The CES-D score obtained for the high job level groups was getting higher as the educational level increased. This is consistent with the study (51) stating that people with higher educational levels felt more depressed according to their CES-D score. The implication of this study was consistent with our results: job-education is an important factor to assess the risk for depression, not just the job or level of education alone.

Lifestyle factors, including physical activity, sleep, and diet, can have a significant impact on depressive symptoms. Regular exercise helps alleviate these symptoms by boosting levels of endorphins, serotonin, and dopamine—neurotransmitters that foster a sense of well-being (52, 53). Sufficient sleep is also essential. Poor sleep quality or inadequate sleep can worsen depression by disrupting the balance of mood-related neurotransmitters and heightening inflammation, including markers like c-reactive protein and systemic inflammation (54, 55). Adopting consistent sleep routines and good sleep hygiene can bolster emotional resilience and lower the risk of depressive episodes. Diet is equally crucial. A well-balanced diet, rich in fruits, vegetables, whole grains, and lean proteins, provides vital nutrients that support brain function (56). Accordingly, future study will need to consider lifestyle as these factors can collectively have a profound impact on mental health, and optimizing them may be key strategy in managing and preventing depression.

In our study, the following limitations should be considered when interpreting the data. First, our data were obtained from KLoSA database which is a self-reported survey. This means that questions about related to CESD score, depressive symptoms, job level, educational level, and job content satisfaction may be affected by subjective responses, false consciousness, and recall bias. This self-reported information about health and symptoms is however critical because it complements objective measures (57). Second, the individual’s ability and characteristics are likely to relate to job level, educational level, and depression. This measure could lead to a potential exaggeration due to a possible failure to include this feature. Third, the education level has grown rapidly in Korea since the Enforcement of the Education Act in 1952. The results of this study may differ in a population composed of highly educated middle-aged and older adults living in OECD countries (58). However, the gap between job level and level of education is still expected to exist, not through the level of education itself about job fitness compared to educational level. Fourth, GEE operates under the assumption that the effect of predictors remains consistent over time, although this may not always hold true. As a result, it may not fully capture associations that fluctuate over time. Finally, although we used longitudinal data, the results may have reflected reverse causality and bidirectional relations in the association between job fitness and depression.

The strength of this study relies on the inclusion of two variables of socioeconomic factors, rather than a simple variable analysis. Unlike previous studies showing that job and education affect depression as independent variables, this study included two factors of socioeconomic level as multi-dimensional variables. Our analysis of the relevance between depression and multiple factors consisted of job level and educational level showed a greater impact on subjects with lower or higher job fitness. Furthermore, we found additional evidence regarding job suitability and job satisfaction and how they were also affected using calibrated variables. Finally, our solid results were obtained considering repeatable targets in the follow-up survey data representative of the Korean population.

In conclusion, unlike previous studies using simple measurements, this study identified the relationship between depression and the gap between job and educational level. This study provides important evidence with great implications for health management and suggests the need to properly monitor the mental health of subjects whose job fitness is unsuitable.

## Data Availability

Publicly available datasets were analyzed in this study. This data can be found here: the dataset analyzed in the present study is publicly accessible. Available online at: https://survey.keis.or.kr/klosa/klosa04.jsp.
